# One-Pot Synthesis of N-Doped NiO for Enhanced Photocatalytic CO_2_ Reduction with Efficient Charge Transfer

**DOI:** 10.3390/molecules28062435

**Published:** 2023-03-07

**Authors:** Fulin Wang, Zhenzhen Yu, Kaiyang Shi, Xiangwei Li, Kangqiang Lu, Weiya Huang, Changlin Yu, Kai Yang

**Affiliations:** 1 School of Chemistry and Chemical Engineering, Jiangxi University of Science and Technology, Ganzhou 341000, China; 2 School of Chemical Engineering, Guangdong University of Petrochemical Technology, Maoming 525000, China

**Keywords:** NiO, nitrogen doping, photocatalysis, reduction of CO_2_

## Abstract

The green and clean sunlight-driven catalytic conversion of CO_2_ into high-value-added chemicals can simultaneously solve the greenhouse effect and energy problems. The controllable preparation of semiconductor catalyst materials and the study of refined structures are of great significance for the in-depth understanding of solar-energy-conversion technology. In this study, we prepared nitrogen-doped NiO semiconductors using a one-pot molten-salt method. The research shows that the molten-salt system made NiO change from p-type to n-type. In addition, nitrogen doping enhanced the adsorption of CO_2_ on NiO and increased the separation of photogenerated carriers on the NiO. It synergistically optimized the CO_2_-reduction system and achieved highly active and selective CO_2_ photoreduction. The CO yield on the optimal nitrogen-doped photocatalyst was 235 μmol·g^−1^·h^−1^ (selectivity 98%), which was 16.8 times that of the p-type NiO and 2.4 times that of the n-type NiO. This can be attributed to the fact that the nitrogen doping enhanced the oxygen vacancies of the NiOs and their ability to adsorb and activate CO_2_ molecules. Photoelectrochemical characterization also confirmed that the nitrogen-doped NiO had excellent electron -transfer and separation properties. This study provides a reference for improving NiO-based semiconductors for photocatalytic CO_2_ reduction.

## 1. Introduction

The rapid development of current society has increased the consumption of non-renewable fossil fuels. Human beings have to face the problem of energy shortages, and the resulting large emissions of CO_2_ are also an important cause of global warming [1,2,3,4]. Currently, using sustainable solar energy to photocatalytically reduce CO_2_ in high-value-added products is a promising way to simultaneously solve the greenhouse effect and the energy crisis [5,6]. Therefore, it is very important to design and synthesize photocatalysts with low pollution, high efficiency, and low cost [7,8].

As an environmentally friendly transition-metal-oxide semiconductor, NiO has excellent conductivity, good chemical stability, and non-toxicity, and it has broad application prospects at the nanoscale [9,10]. At the same time, it is considered to be a semiconductor that can be used for CO_2_ photoreduction due to its sufficiently negative conduction band position, fast hole mobility, and high charge-carrier concentration [11]. However, due to the high recombination degree of photogenerated carriers, the separation efficiency of electrons and holes in the reaction process is low, which greatly weakens the reactivity [12]. In addition, wide-band-gap NiO semiconductor catalysts can only use about 3–5% of solar ultraviolet light, resulting in the low efficiency of the photocatalytic reduction of CO_2_, limiting the application of NiO in photocatalysis [13]. Therefore, NiO is often used as a co-catalyst to improve photocatalytic performance and encourage the efficient separation of photoelectrons and holes [14]. For example, NiO can significantly improve the photocatalytic hydrogen-production performance of SrTiO_3_, TiO_2_, Nb_2_O_5_, Ga_2_O_3_, and other photocatalysts [15]. However, the activity was generally low in the reported photocatalytic reduction of CO_2_ by NiO [16,17]. Therefore, NiO is usually modified by different methods to improve the photocatalytic performance [18].

Since NiO has suitable conduction band (CB) and valence band (VB) positions, it often forms heterostructures with many semiconductors. Zhang et al. [19] prepared an S-type BiOBr/NiO heterojunction. The experiment showed that the layered structure of BiOBr/NiO increased the light-absorption and charge-separation performance, and it improved the redox ability of BiOBr/NiO. In addition, the NiO-layered porous-sheet structure was conducive to the adsorption of CO_2_, exposing abundant active sites for CO_2_ photoreduction, thus achieving excellent CO_2_ photoreduction performance. Moreover, Park et al. [20] prepared a single-layer hollow-sphere photocatalytic material (h-NiO-NiS) of NiO and NiS by partially replacing O with S on NiO hollow spheres. The construction of this heterojunction greatly enhanced the CO_2_-adsorption capacity and increased the transfer of excited electrons from the NiS to the surface along the hollow spheres. The efficient transfer of electrons led to the prolongation of the photogenerated charges’ recombination times, which further increased the conversion of CO_2_ to CH_4_.

Moreover, charge separation can be increased by adjusting the electronic structure of NiO, thereby improving its CO_2_ photoreduction activity. Xiang et al. [21] constructed ultrathin NiO nanosheets with different oxygen-vacancy concentrations to achieve efficient CO_2_ photoreduction performance. Density functional theory calculations and CO_2_-temperature programmed desorption experiments confirmed that moderate oxygen vacancy concentrations achieved a strong combination of the material surface with CO_2_, enhanced the adsorption and activation of CO_2_, and encouraged effective charge transfer. By contrast, the excessive oxygen-vacancy content reduced the binding affinity of the CO_2_; thus, the appropriate regulation of oxygen-vacancy content is an effective means to achieve a NiO electronic structure that is suitable for CO_2_ photoreduction. In addition, the construction of a ternary bridging structure is also an important method to increase the separation of photoelectrons and holes. For example, Park et al. [22] introduced reduced graphene oxide (rGO) into the NiO-CeO_2_ p-n heterostructure, which accelerated the separation and transfer of photogenerated electrons, and the surface of the material accumulated electrons more easily, thus improving the photocatalytic activity of the CO_2_ multi-electron reduction.

In addition, heteroatom doping is an effective method with which to adjust the electronic structures of catalysts and has been extensively studied [23,24,25,26]. However, compared with anion doping, cation doping produces more harmful electron–hole recombination centers. Because oxygen and nitrogen show similar chemical, structural, and electronic characteristics, such as polarizability, electronegativity, coordination number, and ionic radius, when other elements (such as N 2p) with higher potential energy than O 2p atomic orbitals are introduced, new VBs instead of O 2p atomic orbitals can be formed, resulting in smaller E_bg_ without affecting the CB level, thereby improving the visible-light response [27]. Therefore, non-metallic-element-N doping is a preferable way to improve the photocatalytic CO_2_-reduction effect of NiO. Furthermore, it is also important to choose the appropriate doping method. The molten-salt method of element doping is an efficient and low-cost method because its molten-salt liquid environment can make the element distribution more uniform, and the treatment process before and after the reaction is very simple [28].

In this research, NiO semiconductor catalysts with different nitrogen-doping contents were prepared using a molten-salt calcination method, and the CO_2_-reduction activity was tested in a bipyridine ruthenium/triethanolamine heterogeneous catalytic system excited by different wavelengths of light [29]. The phase composition, band structure, optical properties, and surface morphology of the doped NiO semiconductor were researched through a series of characterizations. The enhancement mechanism of the photocatalytic performance was discussed, and the possible mechanism of the photocatalytic process was analyzed. 

## 2. Results and Discussion

### 2.1. Phase Structure

**Figure 1:**
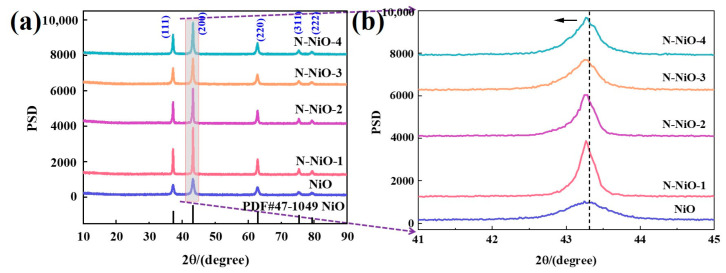
(**a**) The XRD patterns of the prepared samples; (**b**) local amplification diagram.

**Figure 2:**
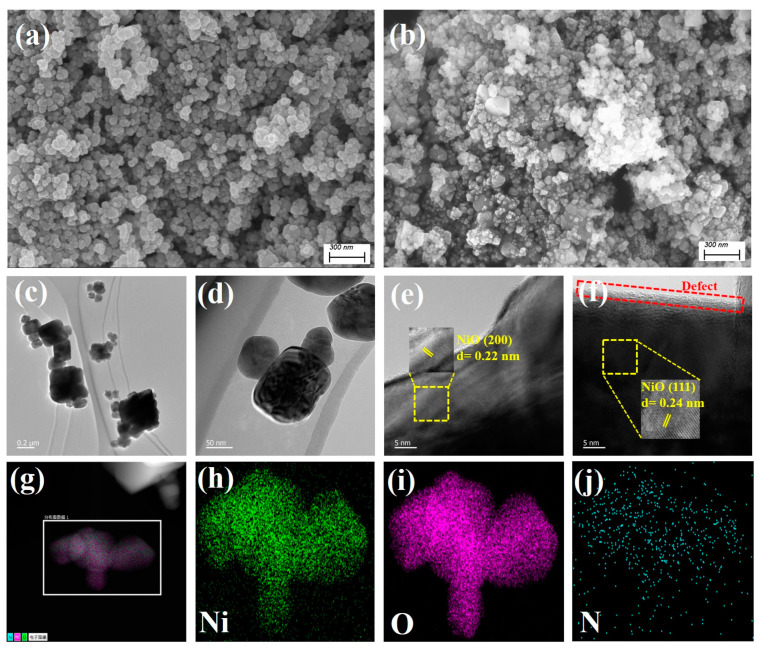
(**a**) SEM images of NiO and (**b**) N-NiO-2; TEM (**c**,**d**), HR-TEM (**e**,**f**), and element-mapping spectra of N-NiO-2 (**g**–**j**).

As shown in Figure 1a, all of the samples corresponded to standard NiO (JCPDS PDF#47-1049), and no impurity phase was detected via XRD. The diffraction peaks at 2θ = 37.2°, 43.3°, 62.9°, 75.4°, and 79.4° corresponded to the (111), (200), (220), (311), and (222) crystal planes of the NiO, respectively [30]. In addition, the doping of the N significantly enhanced the crystallinity of the sample, which was more conducive to the migration and separation of photogenerated charges [31]. By enlarging the range of 2θ = 41–45° (Figure 1b), it was found that the doping of N made the (200) crystal plane of the N-NiO-x shift by a small angle. This is because the radius of the N was different from those of the Ni and the O. After the N doping into the lattice of the NiO, the Ni–O bond became compressed and stretched to a certain extent, resulting in a change in the crystal-plane spacing, which showed the shift in the crystal plane’s diffraction angle macroscopically [32]. The average particle diameters of the samples calculated by the Scherrer equation are shown in Table S1. It was found that the calculated results of the NiO and N-NiO-2 were similar to the results of the SEM (Figure 2a,b). The particle diameter of the pure NiO was smaller and more uniform than that of the N-NiO-2. The doping of the N made the NiO agglomerate and the particle diameter increased.

### 2.2. Microstructure

The microstructure information of the NiO and N-NiO-2 were collected using SEM and TEM. As shown in Figure 2a, the NiO appeared in the form of nanospheres, and the particles were evenly distributed. After the introduction of the N-element doping, the surface of the sample became irregular and agglomerated (Figure 2b). In addition, the TEM images showed that the N-NiO-2 was stacked in sheets and irregularly distributed (Figure 2c), which was similar to the SEM results. Furthermore, as shown by the high-resolution-TEM imagery in Figure 2e–f, it was found that there were lattice-fringe-spacing values of d = 0.22 and 0.24 nm in the N-NiO-2, which corresponded to the (200) and (111) crystal planes of the NiO, respectively. No lattice fringes of impurity phases were detected, indicating that the N doping did not form impurity phases on the surface of the NiO. It is worth noting that the formation of oxygen defects in the sample macroscopically showed the edge of the defect band [33]. In addition, the element-mapping spectra in Figure 2g–j show that the Ni, O, and N elements were uniformly distributed without impurity elements.

### 2.3. Optical Properties

**Figure 3:**
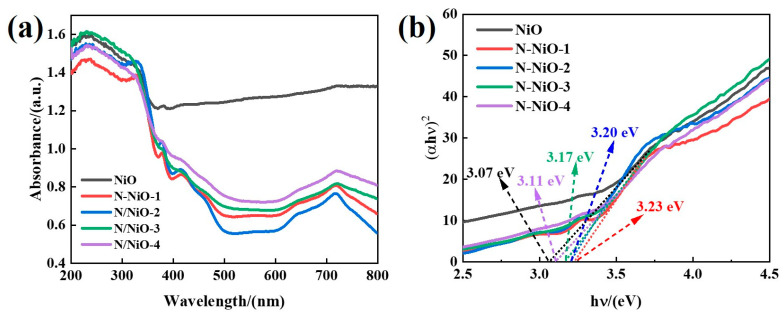
(**a**) Optical absorption properties of samples and (**b**) corresponding (αhν)^2^-hν curves.

The optical absorption spectrum was used to characterize the absorption characteristics of the sample to different wavelengths of light. In general, the larger the maximum absorption wavelength, the wider the spectral response of the semiconductor, but this causes the narrowing of the band gap of the semiconductor, which may further lead to a reduction in the redox performance in the photocatalytic process [34]. Therefore, it was necessary to balance the excitation wavelength and redox performance of the light-excited semiconductor. As shown in Figure 3a, the DRS showed that the maximum absorbance of all the samples was concentrated within a range of 200–350 nm. However, after the N doping, the original black NiO was transformed into yellowish brown N-NiO-x (Figure S1); thus, the absorption of the N-NiO-x in the visible range was weakened. In addition, the absorption peaks of the N-NiO-x at about 390 nm and 470 nm were attributed to the N 2p band introduced by the N doping [35]. The weak absorption band around 600 nm belonged to the defect band [36]. The other absorption peaks at 380–500 nm and the peak around 720 nm correspond to the NiO itself [37].

The band gap of the sample can be calculated according to the Kubelka–Munk equation [38]:

(*αhv*)^1/n^
*=* A(*hv* − *Eg*), (1)

where α is the absorption coefficient, *hv* is the light energy, A is a constant, *Eg* is the band gap, the direct band-gap semiconductor n is 1/2, and the indirect band-gap semiconductor n is 2. According to the literature, NiO is a direct band-gap semiconductor, and n is 1/2. Through drawing a (α*hv*)^2^-*hv* diagram and linearly fitting the curve from the intercept to estimate the Eg of the sample, the results were obtained and they are shown in Figure 3b. It can be seen that the optical absorption of the NiO weakened after the introduction of the N doping into the NiO lattice; on the other hand, the doping of N made the band-gap value of the NiO change from 3.07 to 3.23 eV, and the wider band gap improved the reduction performance of the NiO.

### 2.4. Surface Chemical States

**Figure 4:**
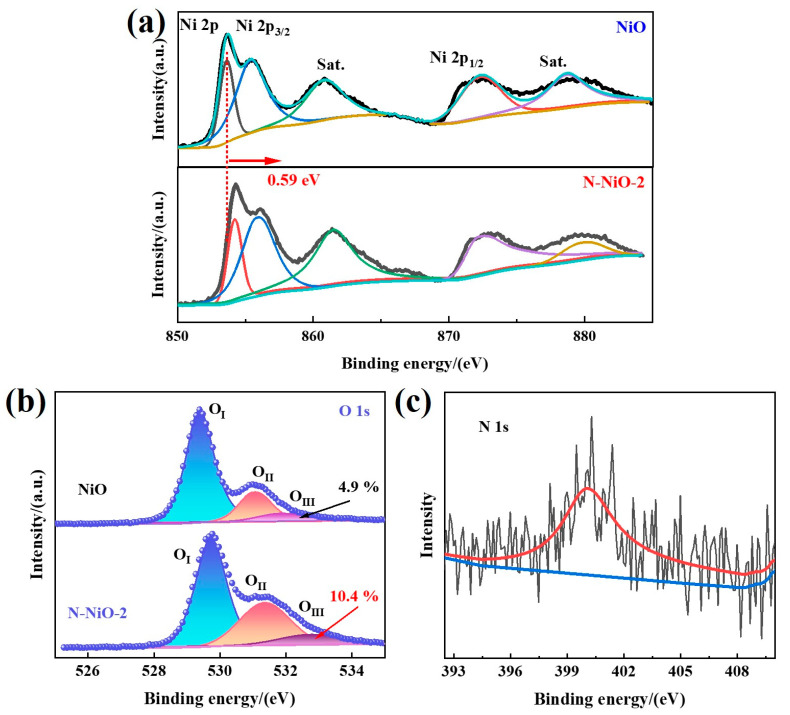
(**a**) X-ray photoelectron spectra of NiO and N-NiO-2: (**a**) Ni 2p, (**b**) O 1s, and (**c**) N 1s.

In the XPS full spectra of the N-NiO-2 shown in Figure S2a, Ni and O elements were present, and no obvious N element was found, which may have been due to the low doping amount. In the C 1s spectrum (Figure S2b), the peaks at 284.8 eV, 286.2 eV, and 288.8 eV corresponded to the C-C, C-O, and C=O of the external carbon source, respectively. Figure 4a corresponds to the energy spectrum of the Ni element. The characteristic peaks of the NiO at the binding energies of 853.6 and 872.0 eV corresponded to Ni 2p_3/2_ and Ni 2p_1/2_, respectively, corresponding to Ni^2+^. In addition, the binding energies of 860.6 and 870.7 eV corresponded to the satellite peaks of Ni 2p [39]. However, compared with the NiO, the Ni 2p characteristic peak of N-NiO-2 shifted 0.59 eV in the direction of increased binding energy, indicating a decrease in the electron-cloud density of the Ni element [40]. This may have been due to the fact that the electronegativity of N is larger than that of Ni, and electrons are more easily attracted by the N element. In addition, it can be seen in Figure 4b that the O 1s were fitted to the three peaks of O_I_, O_II_, and O_III_ with binding energies of 529.2 eV, 531.2 eV, and 531.9 eV, respectively, corresponding to Ni-O lattice oxygen, the hydroxyl oxygen of the adsorbed water on the sample surface and oxygen defects, respectively [41,42,43]. Compared with the NiO, the oxygen defects of the N-NiO-2 increased from 4.9% to 10.4% (Table 1). The increased oxygen defects were more conducive to electron capture, thereby promoting the separation of photogenerated charges [44]. Moreover, the binding energy of 400.0 eV (Figure 4c) corresponded to the N 1s peak, indicating the successful doping of the N element [45]. 

### 2.5. CO_2_-Photoreduction Performance

**Figure 5:**
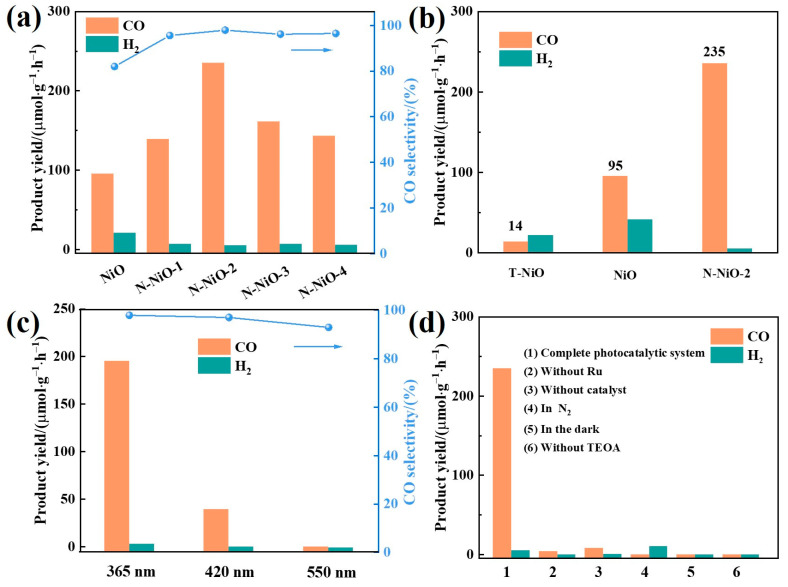
(**a**) Product yields and CO selectivity of NiO and N-NiO-x at 365 nm; (**b**) product yields of T-NiO, NiO, and N-NiO-2 at 365 nm; (**c**) product yield and CO selectivity of N-NiO-2 at different wavelengths; (**d**) product yields of N-NiO-2 under different reaction conditions.

Using a LED lamp as the light source, the prepared samples were tested for CO_2_-photoreduction activities. As shown in Figure 3, it was found by liquid chromatography and gas chromatography that the product had no substances other than CO and H_2_. As shown in Figure 5b, the T-NiO exhibited extremely low CO_2_-reduction activity under 365 nm of light, with a CO yield of 14 μmol·g^−1^·h^−1^ and a selectivity of 39%, while the prepared NiO exhibited higher CO yield (95 μmol·g^−1^·h^−1^) and selectivity (82%) under molten-salt conditions. Furthermore, when N-doping was introduced into the NiO, the CO yield increased to 235 μmol·g^−1^·h^−1^ and the selectivity increased to 98%. As shown in Figure 5a, with the increase in the N content, the yield and selectivity of the CO increased gradually and reached its maximum on the N-NiO-2. In addition, in order to research the photon-utilization rate of the prepared samples, the activity tests were carried out under 420-nanometer and 550-nanometer light sources, and the results are shown in Figure 5c. In order to investigate the necessary conditions of the reaction system in the catalytic process, a control experiment was also carried out. It can be seen from Figure 5d that only trace products were detected under the conditions of no Ru, no catalyst, the use of N_2_ instead of CO_2_, no light, and no TEOA, indicating that the CO did arise from the reduction of CO_2_ in the system, not from the decomposition of the catalyst, and any changes in the reaction system greatly affected the catalytic activity. 

In order to further explore the light-utilization efficiency of the system under the irradiation of different wavelengths of light, the 2-hour CO yield of the N-NiO-2, the calculated apparent quantum efficiency (AQE) value, and the optical power of the corresponding wavelength were determined, and they are listed in Table 2. It can be seen from the table that the AQE reached 2.4% at 365 nm, indicating that the activity corresponded to the energy of the light.

### 2.6. Evaluating the Separation Performance of Photogenerated Carriers

**Figure 6:**
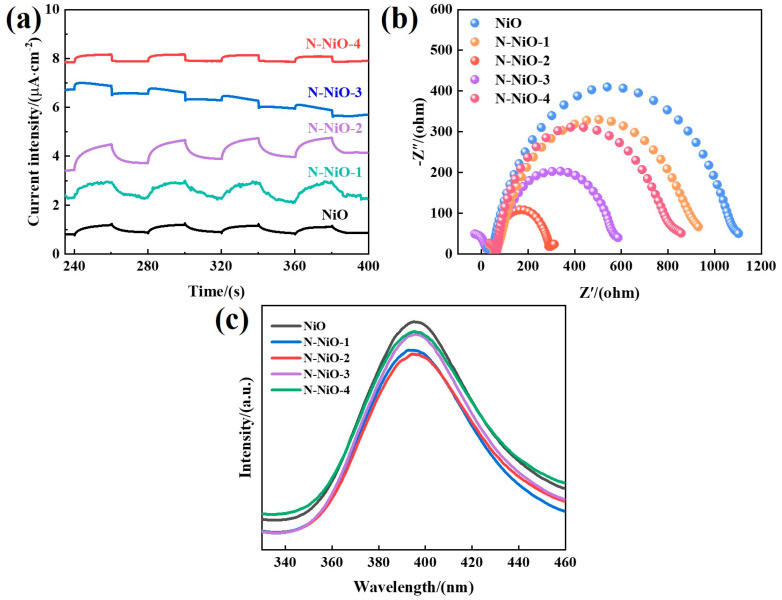
(**a**) Photocurrent-response curves; (**b**) electrochemical-impedance spectra; (**c**) steady-state PL spectra (excitation wavelength: 250 nm).

In order to explore the photogenerated charge-separation abilities of the prepared samples and investigate the resistance during charge transport, photoelectrochemical tests were carried out. As shown in Figure 6a, the N-NiO-x exhibited an enhanced photocurrent response, indicating that the N doping increased the separation of the photogenerated carriers [46]. On the other hand, the electrochemical impedance spectra of the samples (Figure 6b) showed that the doping made the charge-transfer resistance of the NiO smaller, so that the electrons participated in the catalytic reaction more efficiently [47]. In addition, the laws of the photocurrent and the impedance were consistent with the activity law, which also indicated that the charge transfer was the decisive factor in the catalytic activity. Furthermore, the photoluminescence spectra of the prepared samples are shown in Figure 6c. At the excitation wavelength of 250 nm, all the samples showed emission peaks at about 400 nm, which came from the composite luminescence of the photogenerated carriers. The fluorescence-response values of the N-doped samples were weakened, indicating that the degree of recombination of the photogenerated electrons and holes reduced, resulting in more effectively separated electrons, improving the catalytic performance of the photocatalytic reduction system [48].

**Figure 7:**
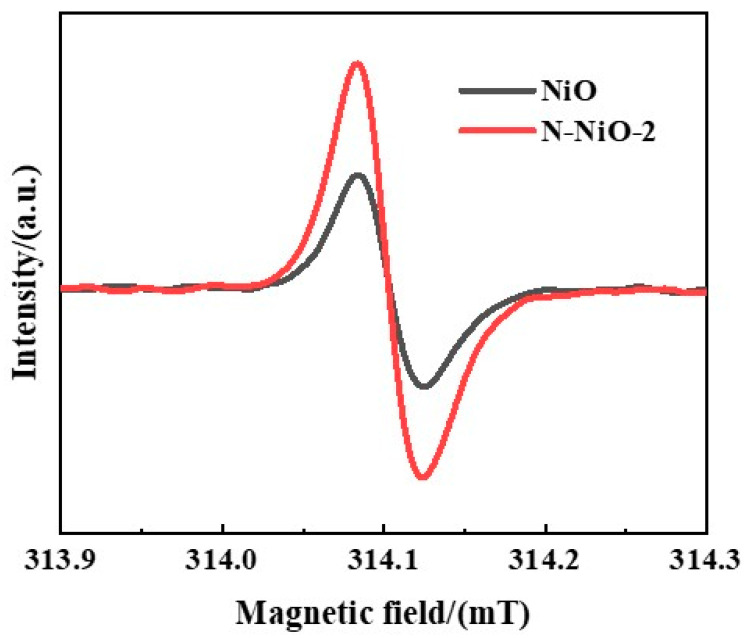
The EPR spectra of NiO and N-NiO-2.

The EPR spectra of the samples at room temperature (Figure 7) revealed the presence of defects in the samples. The Lorentzian linear resonance peaks at g = 2.002 indicated the presence of unpaired electrons in the samples [49,50,51]. The results of the EPR and XPS showed that there were oxygen defects in the samples. The higher Lorentz resonance signal indicated that the N-NiO-2 had more oxygen defects than the NiO, which indicated that the molten-salt system effectively introduced oxygen defects into the NiO, and the presence of N could further increase the formation of oxygen defects. The presence of oxygen defects formed an electron-capture trap in the semiconductor, which encouraged the separation of electrons and holes [52]. The CO_2_ combined with the accumulated electrons in the defects and was reduced to support the improvement in the reaction performance.

### 2.7. Energy-Band Structure

**Figure 8:**
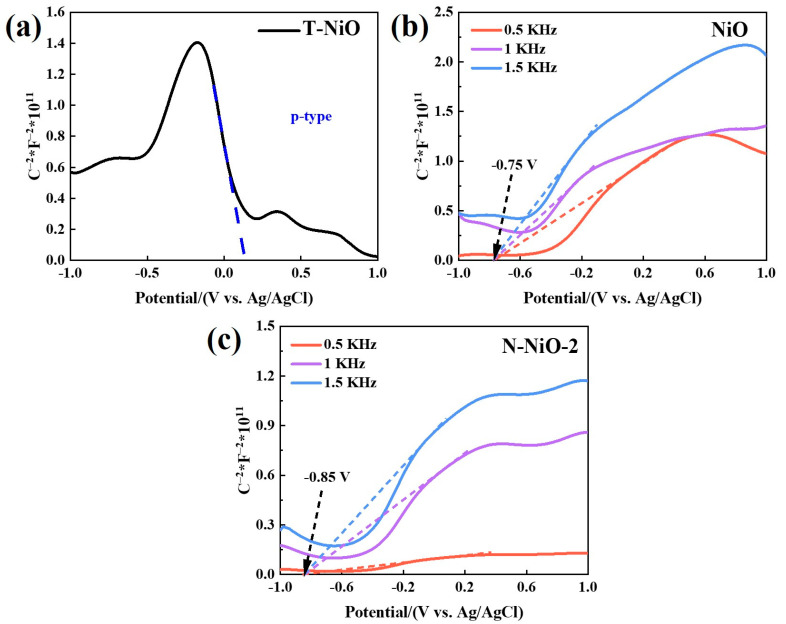
The M–S diagrams of T-NiO (**a**), NiO (**b**), and N-NiO-2 (**c**) semiconductors.

In order to study the energy-band structures and redox properties of the prepared samples, the flat band potentials of the samples were tested via photoelectrochemical Mott–Schottky (M–S) analysis. As shown in Figure 8a, the T-NiO exhibited the characteristics of a p-type semiconductor [53], while the NiO in a molten-salt environment (Figure 8b) exhibited the characteristics of an n-type semiconductor [54], indicating that the molten salt encouraged the transformation of the NiO semiconductor type. The surface of the T-NiO itself was rich in holes, so it showed p-type characteristics. The reduction environment in the molten-salt atmosphere encouraged the formation of oxygen vacancies on the surface of the NiO, further enriching it with surface electrons to realize electron doping. Furthermore, the flat-band potentials of the NiO and N-NiO-2 were −0.75 V and −0.85 V (vs. Ag/AgCl pH = 7), respectively, which corresponded to −0.55 V and −0.65 V (vs. NHE pH = 7), respectively, as shown in Figure 8b,c. In general, the conduction band of the n-type semiconductors was about 0.1 V more negative than the flat-band potential [55], so the conduction band of the N-NiO-2 was reduced from −0.65 V to −0.75 V (vs. NHE pH = 7). This indicates that the N doping reduced the conduction-band position of the NiO and enhanced the reducibility of the reaction. In addition, the valence-band position of the N-NiO-2 was 2.45 V, according to the band-gap diagram obtained through the optical absorption spectrum.

### 2.8. Possible Reaction Mechanism

**Figure 9:**
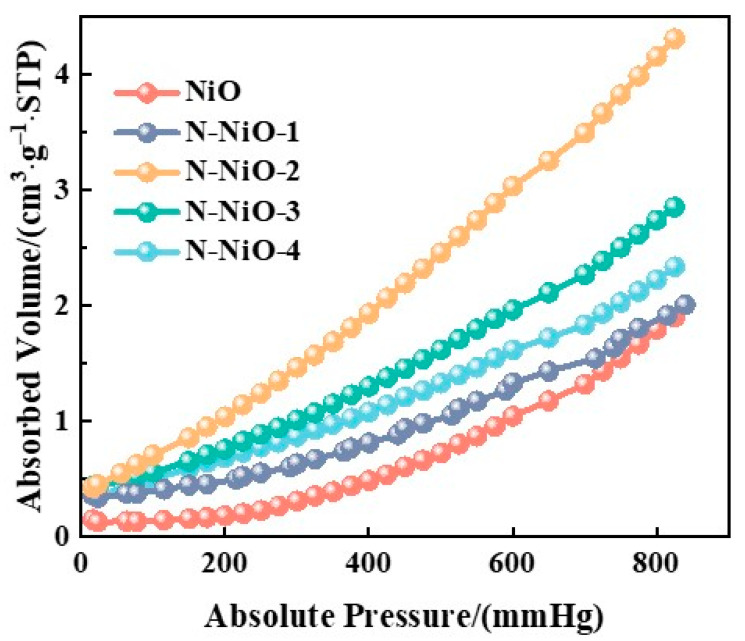
CO_2_-adsorption isotherms of NiO and N-NiO-x.

Figure 9 shows the physical adsorption isotherms of the CO_2_ on the prepared samples. Compared with the pure NiO, the adsorption capacity of the N-NiO-x materials for the CO_2_ increased first and then decreased with the increase in the N content, and it reached a maximum with the N-NiO-2 sample, which was consistent with the order of reactivity. These results show that the N doping increased the adsorption of CO_2_ on the surface of the NiO, and the combination of electrons with CO_2_ on the surface of the NiO facilitated the photoreduction performance of the CO_2_, indicating that the adsorption of CO_2_ was the decisive factor in the activity. 

**Figure 10:**
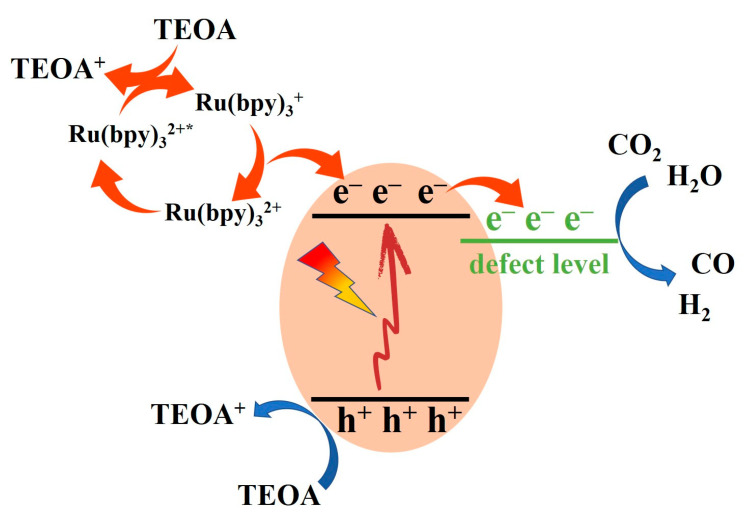
Possible photoreduction mechanism of CO_2_ on N-NiO-x (* represents the excited state).

The possible mechanism of the whole reaction is shown in Figure 10. Under illumination, the N-NiO-x semiconductor became excited, and it produced electron–hole pairs (e^−^–h^+^). Subsequently, the excited electrons in the conduction band of the N-NiO-x transferred to the defect energy level and accumulated there. The holes accumulated in the valence band were consumed by triethanolamine (TEOA), and the TEOA oxidized to diethanolamine and glycolaldehyde. At the same time, Ru(bpy)_3_^2+^ activated to the excited state of Ru(bpy)_3_^2+*^ under light irradiation and was then quenched by the TEOA to form Ru(bpy)_3_^+^. Subsequently, the electrons of the Ru(bpy)_3_^+^ were transferred to the conduction band of the NiO, and further accumulated at the defect level, and the Ru(bpy)_3_^+^ was oxidized to the initial state, Ru(bpy)_3_^2+^. Furthermore, CO_2_ molecules combined with the excited-state electrons accumulated at the N-NiO-x defect level and protons in water and converted into the product, CO. 

## 3. Experimental Section

### 3.1. Materials

The used chemicals were nickel nitrate hexahydrate (Ni(NO_3_)_2_·6H_2_O, Sinopharm Chemical Reagent Co., Ltd., Shanghai, China), sodium hydroxide (NaOH, Shanghai Aladdin Biochemical Technology Co., Ltd., Shanghai, China), anhydrous lithium chloride (LiCl, Shanghai McLean Biochemical Technology Co., Ltd., Shanghai, China), potassium chloride (KCl, Xilong Science Co., Ltd., Shantou, China), urea (CH_4_N_2_O, Shanghai McLean Biochemical Technology Co., Ltd., Shanghai, China), [Ru(bpy)_3_]Cl_2_·6H_2_O(Shanghai McLean Biochemical Technology Co., Ltd., Shanghai, China), triethanolamine (TEOA, Xilong Science Co., Ltd., Shantou, China) and acetonitrile (MeCN, Xilong Science Co., Ltd., Shantou, China). All chemicals were analytically pure and could be used directly without purification after purchase. 

### 3.2. Synthesis of Precursor Ni(OH)_2_

Precursor Ni(OH)_2_ was prepared by a simple precipitation method: a total of 10 mmol Ni(NO_3_)_2_·6H_2_O was dissolved in 40 mL of deionized (DI) water under magnetic stirring, after which 20 mmol of NaOH was added when the solid was completely dissolved. After 30 min of stirring, the precipitate was collected by filtration, washed once with 10 mL of deionized (DI) water and anhydrous ethanol, and dried at 60 °C for 8 h to obtain precursor Ni(OH)_2_. 

### 3.3. Synthesis of NiO

The 5 mmol of precursor Ni(OH)_2_ was fully ground with 2.7 g of LiCl and 3.3 g of KCl and then calcined at 400 °C for 3 h. After the reaction, the bulk was fully dissolved in DI water and filtered, after which it was washed several times with DI water and ethanol alternately before drying at 60 °C for 8 h to obtain NiO. For comparison, traditional P-type NiO (T-NiO) was obtained by directly calcining Ni(OH)_2_.

### 3.4. Synthesis of N-NiO-x

With urea as the nitrogen-doping source, excessive urea was added to the reaction to reduce the effect of volatilization. The preparation process of nitrogen-doped NiO was as follows: a total of 5 mmol of precursor Ni(OH)_2_, m g urea (m = 0.2,0.3,0.4,0.5), 2.7 g of LiCl, and 3.3 g of KCl were fully ground and then calcined at 400 °C for 3 h. The bulk after reaction was fully dissolved in appropriate DI water and filtered, after which it was washed several times with DI water and ethanol alternately and dried at 60 °C for 8 h to obtain N-NiO-x (x is 1, 2, 3, 4). Nitrogen and oxygen contents over N-NiO-2 were determined by inert-gas-fusion technique using a nitrogen-and-oxygen elemental analyzer (LECO Corp., TC-436AR, St. Joseph, USA). The carbon content was obtained by carbon–sulfur analyzer. The ratio of C over N-NiO-2 was 0.02 wt% (probable error), which was negligible compared with 2.03 wt% N and 19.21 wt% (Table S2). 

### 3.5. Photocatalytic CO_2_ Reduction

In this research, the catalytic performances of the samples were evaluated for CO_2_-reduction activity. The light source was an 80-watt LED lamp (illumination wavelengths were 365 nm, 420 nm, 550 nm, Zhenjiang Yinzhu Chemical Technology Co., Ltd., Zhenjiang, China). Typically, 30 mg of the catalyst, 5 mg of [Ru(bpy)_3_]Cl_2_·6H_2_O (denoted as Ru), 3 mL of MeCN, 2 mL of H_2_O, and 1 mL of TEOA were added to a 50-milliliter quartz reactor. Before the start of the reaction, the reactor was first vented with pure CO_2_ for 30 min in the dark, in order to make the reaction system reach the adsorption saturation of CO_2_; next, 1 mL of gas was extracted every 2 h under illumination and injected into a chromatographic system (H_2_ and CO were detected by thermal-conductivity detector and flame-ionization detector, respectively).

The CO selectivity was calculated using the following formula:

(2) $$\mathrm{CO}\;\mathrm{Selectivity}\;(S_{\mathrm{CO}})=\;\frac{Y_{CO}}{Y_{CO}+Y_{H2}}\;\;$$

where *Y_CO_* and *Y_H_*_2_ represent the yields of CO and *H*_2_, respectively.

Furthermore, the optical powers at different wavelengths were measured via an optical power meter, with a probe area of 1 × 1 cm^2^ to contact light. The light-irradiation area was 2.5 × 2.5 cm^2^. The apparent quantum efficiency (AQE) was calculated using the following formula:

(3) $$AOE=\frac{2\times the\;number\;of\;evolved\;CO\;molecules}{N}$$

(4) $$N=\frac{E_{\lambda }}{hc}\;$$

where

*N*: the number of incident photons;

*E*: the accumulated light energy in the given area (J);

 λ: the wavelength of the light;

*h*: Planck’s constant (6.626 × 10^−34^ J·s);

*c*: the velocity of light (3 × 10^8^ m·s^−1^).

### 3.6. Characterizations

The phase structure of the material was measured using X-ray diffraction (XRD, Cu *Kα*, *λ* = 0.15406 nm, Bruker D8 Advance). The microstructure and element distributions of the prepared samples were evaluated using scanning-electron microscopy (SEM, FESEM ZEISS sigma 500, Oberkochen, Batenwerburg, GER), transmission-electron microscopy (TEM, JEM-2100F), and energy-dispersive X-ray spectroscopy (EDX). The X-ray photoelectron spectra (XPS, Thermo Fisher, K-Alpha, Waltham, MA, USA) were examined to study the chemical states of the elements. The UV–Vis diffuse-reflectance spectra (DRS, Shimadzu UV-2600, Kyoto, Japan) were examined using BaSO_4_ as the reference standard, in order to study the optical absorption properties of the samples. The vacancy-defect state in the photocatalyst was analyzed with electron paramagnetic resonance (EPR, Bruker ER200-SLC, Billerica, MA, USA) measurement at room temperature. The CO_2_ adsorption at 273 K under ice–water-mixture conditions was studied on an automatic physical adsorption instrument (ASAP 2020, Norcross, Georgia, USA). Steady-state fluorescence (PL) spectra detected the reintegration of exposed electron–hole pairs at an excitation wavelength of 250 nm with a fluorescence spectrometer (FLS 980, Edinburgh, Scotland). Photoelectrochemical measurements were carried out in a three-electrode system on an electrochemical workstation (Shanghai Chenhua CHI-660E, Shanghai, China) using 0.1 mol/L Na_2_SO_4_ or 0.1 mol/L K_3_Fe(CN)_6_/K_4_Fe(CN)_6_ buffer solution as the electrolyte solution, Ag/AgCl as the reference electrode, Pt wire as the auxiliary electrode, and indium-tin-oxide conductive glass (ITO) as the working electrode (10 mg of the sample was dissolved in 3 drops of ethanol, including 10 μL of nafion solution, after which the solution was subjected to ultrasound for 40 min to completely disperse the sample, with an effective loading area of 0.25 cm^2^). 

## 4. Conclusions

In summary, NiO semiconductors doped with non-metallic nitrogen were successfully prepared using a molten-salt method. Compared with the p-type NiO, the reduction performance of the n-type NiO was improved. Furthermore, the photoreduction of CO_2_ by the n-type NiO was more efficient with N doping. The improvement in the photocatalytic performance of the NiO semiconductor doped with non-metallic nitrogen was mainly due to three factors: (1) the molten-salt atmosphere increased the transformation of the p-type NiO to n-type and the conduction-band position met the potential requirements for CO_2_ reduction, thus enhancing the reduction performance; (2) the nitrogen doping increased the adsorption and activation of CO_2_ on the surface of the NiO semiconductor, and realized the rapid conversion of CO_2_; 3) the defect-energy level induced by the oxygen defects increased the transfer and separation of electrons, and the CO_2_ obtained electrons at the oxygen defects more easily and reduced. This research provides a new reference for solving the insufficient reduction performance of p-type NiO, as well as a new control method for inhibiting the photogenerated charge recombination of n-type NiO semiconductors. 

## Figures and Tables

**Table 1:** **The** XPS fitted peak area and oxygen-defect ratios on NiO and N-NiO-2.

	Oxygen Species	Ni–O	O–H	OV	OV Ratio
Samples					
NiO		64,587	44,182	5635	4.9%
N-NiO-2		67,586	73,293	16,352	10.4%

**Table 2:** Optical power, CO yield, and AQE of the N-NiO-2 at different wavelengths under 2 h of illumination.

Wavelength (nm)	Optical Power (mW)	CO Yield (μmol·g^−1^·h^−1^)	AQE (%)
365	456.4	235.5	2.4
420	358.7	48.2	0.5
550	59.2	1.3	0.06

## Data Availability

Not applicable.

## Supplementary Materials

The following supporting information can be downloaded at: https://www.mdpi.com/article/10.3390/molecules28062435/s1, Table S1: Average particle diameters of the samples; Table S2: The contents of C and N obtained by carbon-sulfur analyzer and nitrogen-oxygen elemental analyzer; Figure S1: Photos of NiO and NiO-x; Figure S2: XPS survey spectra (a) and C 1s spectra (b) of N-NiO-2; Figure S3: TCD (a) and FID (b) of gas chromatogram; (c) Liquid chromatogram.

## Sample Availability

Samples of the compounds are not available from the authors.
