# Supplementary material for: One-Pot Synthesis of N-Doped NiO for Enhanced Photocatalytic CO2 Reduction with Efficient Charge Transfer
Source: Molecules. 2023 Mar 7;28(6):2435. doi: 10.3390/molecules28062435 (PMC10057620; doi:10.3390/molecules28062435)
Supplement: Supplementary file 1 [file molecules-28-02435-s001.zip › molecules-2152270-supplementary.pdf]

Supporting Information

One-pot Synthesis of N-doping NiO for Enhanced Photocatalytic CO_2 Reduction with Efficient Charge Transfer

1. Particle diameters

The particle diameter of the sample was obtained using the Scherrer equation:

$$D = \frac{K\lambda}{\beta \cos\theta}$$

where,

D = particle diameter (nm)

K = 0.9 (Scherrer constant)

λ = 0.15406 nm (wavelength of the x-ray sources)

β = FWHM (radians)

θ = peak position (radians)

Finally, the average particle diameter was calculated, and the results were shown in the Table S1.

Table S1 Average particle diameters of the samples

Samples	NiO	N-NiO-1	N-NiO-2	N-NiO-3	N-NiO-4
Particle diameter (nm)	12.0	23.6	22.5	15.4	15.5

2. Element contents

Table S2 The contents of C and N obtained by carbon-sulfur analyzer and nitrogen-oxygen elemental analyzer

Samples	C (wt%)	N (wt%)	O (wt%)
N-NiO-2	0.02	2.03	19.21

3. Photos of NiO and NiO-x

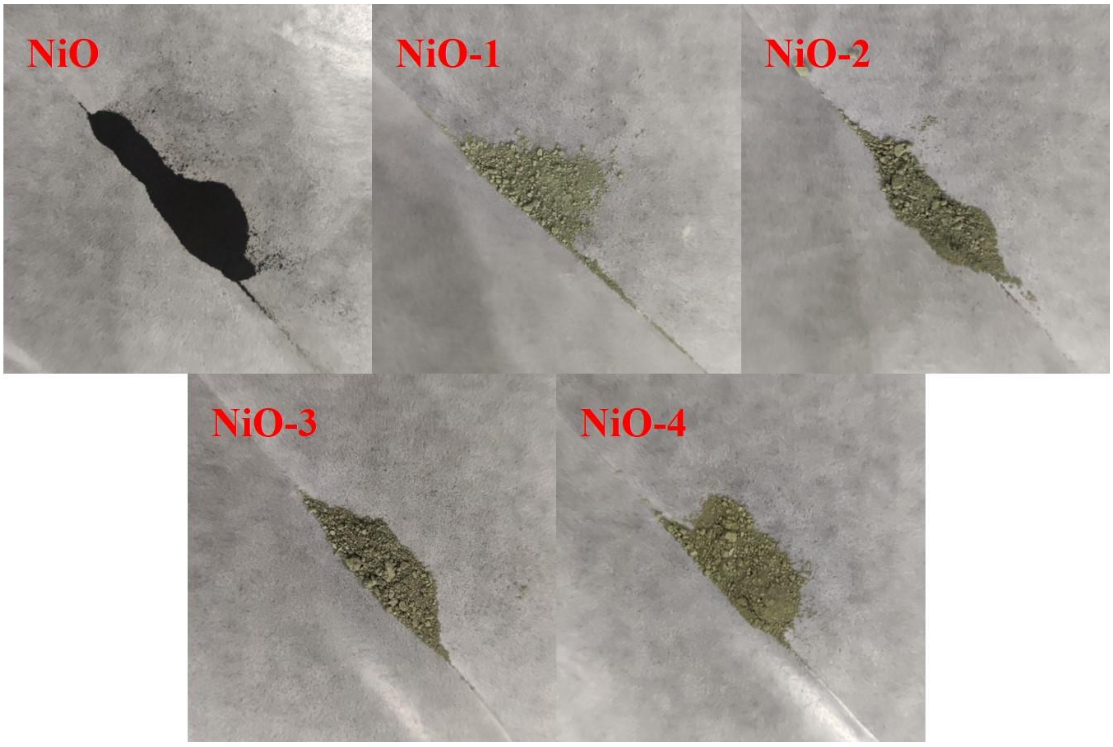

Figure S1. Photos of NiO and NiO-x.

4. XPS

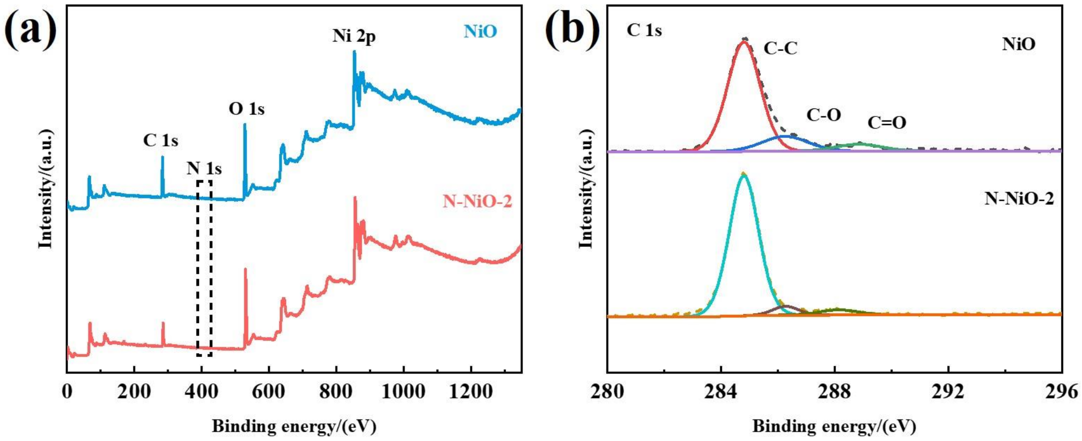

Figure S2. XPS survey spectra (**a**) and C 1s spectra (**b**) of N-NiO-2.

5. Chromatogram

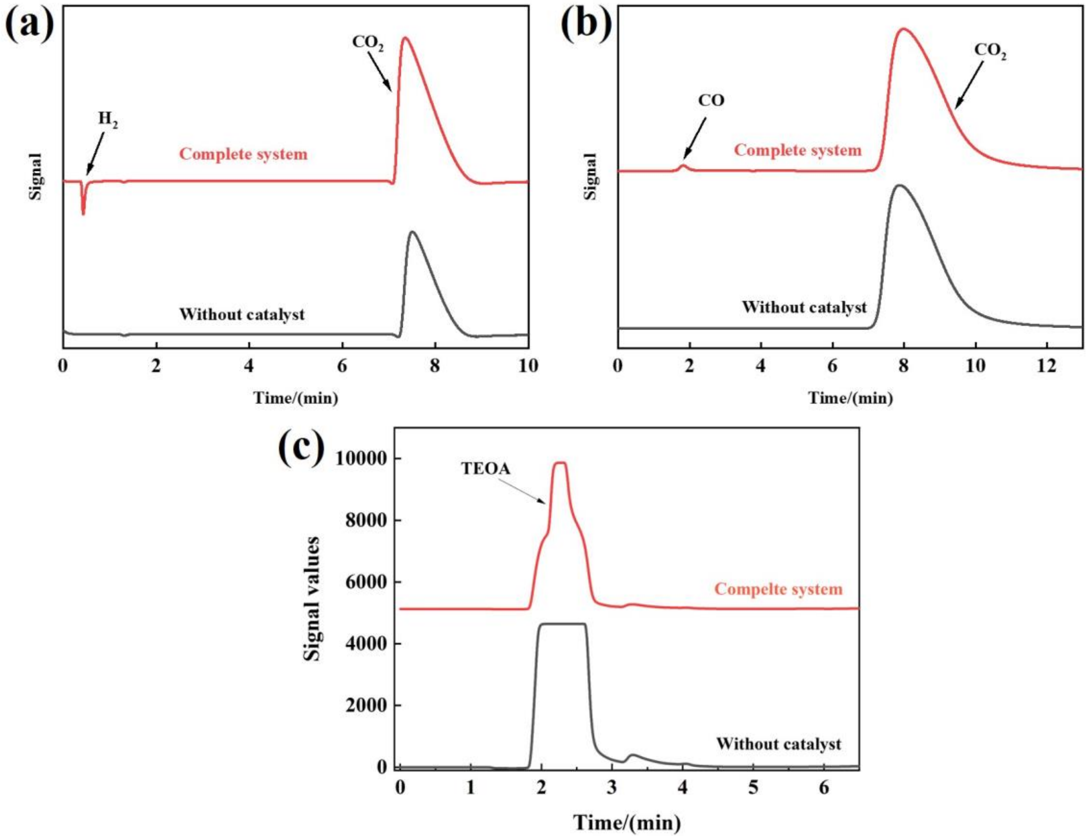

Figure S3. TCD (**a**) and FID (**b**) of gas chromatogram; (**c**) Liquid chromatogram.
